# Clinical validation of MR‐generated synthetic CT by MRCAT for brain tumor radiotherapy

**DOI:** 10.1002/acm2.14494

**Published:** 2024-12-13

**Authors:** Tyrone Tsz Yeung Yip, Zhichun Li, Tian Li

**Affiliations:** ^1^ Department of Health Technology and Informatics Faculty of Health and Social Sciences The Hong Kong Polytechnic University Hong Kong Hong Kong

**Keywords:** brain tumor, MRCAT, MRI, MR‐only radiotherapy, radiotherapy, synthetic CT

## Abstract

**Objective:**

MRI is an emerging modality in radiotherapy (RT). Accuracy synthetic CT is the prerequisite for implementing MR‐only RT planning. This study validated the commercial algorithm of MR for calculating attenuation (MRCAT) in terms of image quality and dosimetric agreement.

**Methods:**

Brain tumor cases with 18 treated using intensity‐modulated radiotherapy (IMRT) or volumetric modulated arc therapy (VMAT), and 15 treated using stereotactic radiosurgery (SRS) were analyzed. Synthetic CTs were resampled referencing planning CT. Treatment plan calculated on planning CT was recalculated on resampled MRCAT. Image quality of selected metrics and dosimetric agreements were assessed by dose‐volume‐histogram and 3D gamma analysis.

**Results:**

For IMRT/VMAT and SRS cases, mean error were 23.42 ± 1.05 and 28.39 ± 3.17 HU; mean absolute error were 38.03 ± 1.42 and 52.36 ± 2.63 HU; root mean squared error were 89.09 ± 6.65 and 108.38 ± 12.23 HU; peak signal‐to‐noise ratio were 29.11 ± 0.60  and 27.65 ± 0.59 dB; and structural similarity index measures were 0.88 ± 0.00 and 0.70 ± 0.01 respectively. No significant differences were identified for DVH metrics accounting the target coverage. Most OARs did not have significant dose deviation, except left lens with 0.70% higher in D‐mean after recalculation (*p* < 0.001). For criteria of 3 mm/3%, 2 mm/2%, and 1 mm/1%, gamma passing rates for IMRT/VMAT were 99.92%, 99.42%, and 96.47%, while SRS were 99.86%, 99.52%, and 97.57% respectively. Correlation between passing rate and image quality metrics was established in IMRT/VMAT cases, with higher similarity yield better dosimetric agreement between planning and synthetic CT.

**Conclusion:**

This study has validated the MRCAT for clinical use in terms of comparable image quality and dosimetric agreement with planning CT. Further case selection and MR‐compatible immobilization device would be required.

## 1 INTRODUCTION

Neoplasm of brain has been a significant contributor to human malignancies. Radiotherapy (RT) has provided non‐invasive access to deep‐seated tumors and diminished the reliance on patient compliance as in surgery and chemotherapy, and thus, is a major component in brain tumor management.^1^, ^2^ Benefited from the invention of multi‐leaf collimator (MLC), intensity modulation has been adopted for most adjuvant treatment of high‐grade tumors or sub‐total resection using intensity‐modulated RT (IMRT) or volumetric‐modulated arc therapy (VMAT), and palliation of small and limited number of brain metastasis using hypofractionated stereotactic RT (SRT) or stereotactic radiosurgery (SRS). As inverse planning is adopted, modulating beamlets fluence across fields by MLC is possible for creating sharp dose gradient, allowing dose escalation, while reducing margin to preserve adjacent normal tissues. However, tolerance to error in localization and setup is also reduced.^3^ While the latter one could be controlled by rigid immobilization and image verification, accurate target delineation and organ‐at‐risk (OAR) contouring requires simulation images with high spatial, contrast resolution, and functional information.

MRI is an emerging modality for RT simulation. While the acquisition uses non‐ionizing radiation, it also provides superior contrast resolution for better visualization of interface between tumor and normal parenchyma,^4^, ^5^, ^6^ which is important for intra‐cranial region with fine anatomical structures and complex cellular compositions. The capability of functional imaging also supplemented bio‐information to anatomical images,^4^, ^5^ like tumors of restricted diffusivity could be differentiated from adjacent tissues under the diffusion‐weighted imaging (DWI). Despite the benefits offered, MRI lacks electron density for dose calculation, as image contrast is dependent on acquisition parameters in MR sequences.^4^, ^5^ The geometric distortion by field inhomogeneity and gradient non‐linearity also affects the accuracy of planning and subsequent dose delivery.^4^, ^5^, ^6^ Increased acquisition time compared to CT might also be burdensome to paediatric or palliative cases of poor compliance.^4^, ^5^

In the view of limitations, MR‐simulation is mostly supplemented to CT‐simulation by registration for delineation, known as MR‐CT pathway.^4^, ^5^, ^6^ While increasing clinical burden with extra resources, this also introduced registration error ranging from 0.5  to 3.5 mm, affecting the accuracy of planning. Recent workflow has proposed the exclusive adoption of MR‐simulation throughout the planning procedures to eliminate registration procedures, known as MR‐only pathway. Target and OAR contouring would be performed on MR‐simulation images, and structure sets would then be transferred to a set of synthetic CT generated from the respective MR‐simulation images using preset model or algorithms for dose calculation. Such approach is dependent on quality of synthetic CT algorithms. Despite significant progresses made, most synthetic CT algorithms are under developmental stage, in which further validation and refinement are required.

Conventionally, synthetic CT is generated by model‐based algorithms.^4^, ^7^ Earliest technique adopted bulk density override by assigning homogeneous water equivalent density to entire body volume. Since discrepancies up to 2% were recorded from dose calculated on planning CT with tissues heterogeneity,^7^, ^8^, ^9^ tissue segmentation was added, followed by bulk density override.^10^, ^11^ Despite the improvement, manual segmentation for each patient is infeasible for clinical practice. Atlas‐based technique register newly acquired MRI to MR‐atlas in the database and transfer the resultant deformation vector field (DVF) to the co‐registered CT atlas for synthetic CT generation.^12^, ^13^ However, anatomical variation across patients is challenging due to distorted shape of tumor and adjacent structures. Voxel‐based technique achieved tissue classification by thresholding MRI images, including T1 and T2*‐weighted sequences for soft tissue,^14^, ^15^ ultrashort echo time (UTE) or zero echo time (ZTE) for bone with short T2,^16^, ^17^ or DIXON sequence based on chemical shifting properties.^18^, ^19^

Recent progress in artificial intelligence (AI) has provided insight for deep‐learning approach.^20^ Convolutional neural network (CNN) constructed with multiple processing layers are often adopted to train synthetic CT algorithm under regulations of voxel‐wise loss functions, which is a similarity index accounting the difference of generated images to ground truth planning CT, and repeating the generation until acceptable similarity is achieved. MR for Calculating ATtenuation (MRCAT) provided by the Philips (Philips Healthcare, Best, The Netherlands) is a commercial example. Tissue classification would be performed on source MRCAT, which are two sets of in‐phase and water DIXON MRI images from compressed SENSE accelerated dual echo T1‐weighted GRE 3D mDIXON sequence with fixed acquisition parameters. Subsequent bone and body outline segmentation followed by HU assignation for bone and soft tissue would then be conducted by AI‐trained algorithm to generate synthetic CT automatically. Despite being approved by Food and Drug Administration (FDA), most studies concerning MRCAT or other commercial algorithms are dedicated for pelvic region,^21^, ^22^ validation data of brain region are still lacking. This study aims to perform a clinical validation on the MRCAT Brain by image quality and dosimetric agreement with planning CT, to evaluate its potential implementation into MR‐only RT workflow.

## 2 METHODS

### 2.1 Patient data

This study evaluated 33 cases of brain tumors treated with RT between July 2022 and June 2023. Prescriptions included 40.05 Gy over 15 fractions, and 54  or 60 Gy delivered over 30 fractions for radical IMRT/VMAT; and 18.75, 22.5, or 27.5 Gy at different isodose level (IL) within single fraction for palliative SRS. Sampled data for each case included one set of planning CT with structure sets and treatment plans, and two sets of source MRCAT of in‐phase and water image, and one set of corresponding synthetic CT. All planning target volume (PTV) were delineated by oncologists, and OARs were contoured by radiation therapists. Treatment plans were computed by medical physicists or radiation therapists. Cases with motion or susceptibility artifacts within intra‐cranial region of simulation images were excluded.

### 2.2 CT‐simulation and MR‐simulation protocol

**FIGURE 1:**
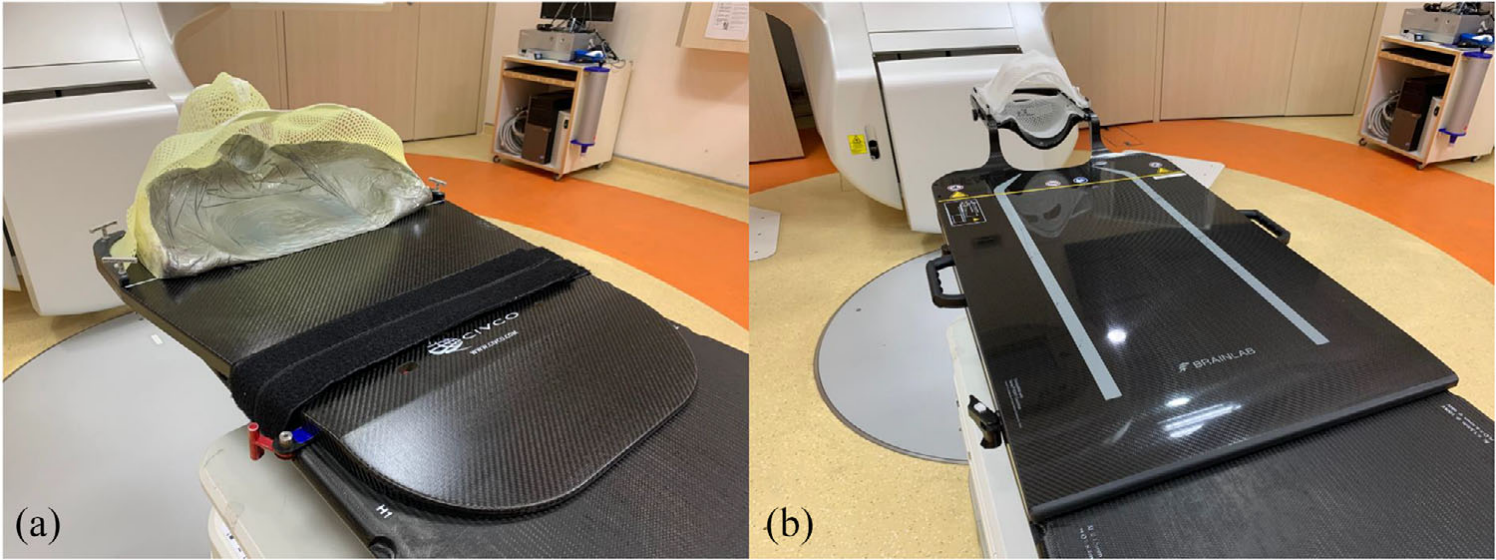
Treatment setup illustration. Setup for IMRT/VMAT (a). Patients were lied supine with customized headrest, thermoplastic cast mounted on Type‐S frame and locked on Overlay Board. MR‐compatible version is available. Setup for SRS (b). Patients were lied supine with three‐layered thermoplastic cast mounted on Cranial 4pi Stereotactic Mask and locked on the dedicated baseplate. MR‐compatible version is not available.

Head‐in supine position was adopted for all cases. For IMRT/VMAT cases, CT‐simulations were performed with thermoplastic cast mounted on Type‐S and Overlay Board (Civco Radiotherapy, Iowa, USA) as illustrated in Figure 1a. The setup was reproduced using MR‐compatible version of Type‐S immobilization system in MR‐simulation. Whereas for SRS cases, CT‐simulation used the Cranial 4pi Stereotactic Mask (BrainLab, Munich, Germany) with overlay as demonstrated in Figure 1b, while supine with dedicated pillow under head were adopted to achieve comfortable neck position for scanning due to the unavailability of MR‐compatible version of stereotactic mask.

CT‐simulations were performed in a 16‐slice version of brilliance big bore CT simulator (Philips Healthcare, Best, The Netherlands), with consistent tube voltage of 120 kVp for matching the calibration curve in treatment planning system (TPS). The field on view (FOV) of images were 515 × 512 pixels with 0.684 mm spacing between pixels. The slice thickness varied with the RT technique. For VMAT/IMRT cases with locoregional size of target, slice thickness was 3 mm. For SRS cases treating small‐sized lesion, slice thickness of 1.5 mm was chosen.

MR‐simulations were conducted in a 1.5 T ingenia ambition MR simulation system (Philips Healthcare, Best, The Netherlands) with flexible coils, using MR sequences from the departmental brain region simulation protocol: fast field echo (FFE) survey for planning the FOV of the scan; T2‐weighted axial turbo spin echo (TSE) for pathological screening of brain region; dual‐echo 3D FFE mDIXON MRCAT for capturing chemical shift differences to generate source images for synthetic CT synthesis; and post‐contrast T1‐weighted axial FFE for intra‐cranial lesions and metastasis detection. Detailed acquisition parameters for each sequence are listed in the Table 1.

**TABLE 1:** MR‐simulation protocol.

Sequences information			Acquisition settings
T1 Survey	Sagittal	2D FFE	FH × AP × RL = 300 × 300 × 50 mm Voxel size = 0.98 × 1.95 × 10 mm^3^ NSA = 1, TR/TE = 15 ms/Shortest BW = 186.6 Hz, FA = 20
T2	Axial	2D TSE	AP × RL × FH = 230 × 182 × 150 mm Voxel size = 0.75 × 0.94 × 3 mm^3^ NSA = 2.5, TR/TE = 5005/100 ms BW = 170.5 Hz, FA = 90, TSE Factor = 15
MRCAT	Axial	3D FFE mDIXON	AP × RL = 248 × 232 Voxel size = 1.11 × 1.10 × 1.40 mm^3^ TR/TE = 6.8/2.0/4.4 ms BW = 481 Hz, FA = 20
T1 post‐contrast	Axial	3D FFE	AP × RL × FH = 230 × 178 ×170 mm Voxel size = 1 × 1 × 1 mm^3^ NSA = 2, TR/TE = 25/6.1 ms BW = 189.7, FA = 30

Abbreviations: BW, bandwidth; FA, flip angle; FFE, fast field echo; NSA, number of signal average; TE, echo time; TSE, turbo spin echo; TR, repetition time.

### 2.3 Data pre‐processing

While treatment position was reproduced, there were still inconsistences between the planning and synthetic CT, inducing uncertainties for data analysis. First, synthetic CT was generated from MRI that cannot capture signal intensities of the couch top and immobilization devices as in CT. Thus, error would be induced during image quality comparison and dose calculation based on HU of pixels. Second, positioning difference might be induced between planning CT and synthetic CT. Since images are acquired in separated sessions, repositioning error tends to happen in the neck position, tilting angle of head, and particularly for facial contours in SRS cases due to lack of the stereotactic mask during MR‐simulation. Third, organ motions and disease progressions might happen between sessions of simulations. For brain tumor cases, mouth bites were not indicated as a part of immobilization since it is distant from the region of interest (ROI). This might cause difference in tongue motion across simulation sessions. Therefore, following pre‐processing were performed to minimize confounding listed and increased the internal validity of further analysis.

#### 2.3.1 Mould and treatment accessories removal for planning CT

**FIGURE 2:**
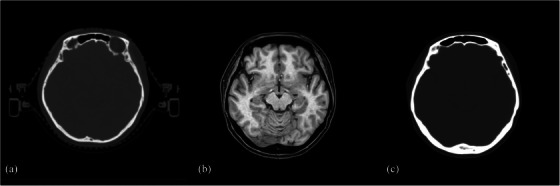
Axial image for a subject with reproduced setup in both CT‐ and MR‐simulation. Signal intensity of immobilization device is present in Planning CT (a), while absent in Source MRCAT (b) and MRCAT (c).

The synthetic CT generated from source MRCAT lacked HU from the MR‐compatible couch and all treatment accessories as illustrated in Figure 2, impacting the accuracy of subsequent data analysis. Since adding treatment accessory structures with assigned HU to synthetic CT in the TPS might induce extra uncertainties and require calibration of MR‐compatible couch and instruments, this study has eliminated HU outside body contour in CT‐simulation images for data analysis.

The removal procedures were performed to all planning CT. DICOM images were first loaded to the 3D Slicer (Slicer Community, Massachusetts, USA), and displayed all CT slices with contoured structure sets extracted from the TPS. After that, the contours of “BODY” for each case were converted to binary vector volume and exported to respective files. Resultant binary masks and planning CTs were then overlaid with the same origin, and pixels outside the binary mask were assigned with zero HU using in‐house code created in the MATLAB (MathWorks, Massachusetts, USA), while the HU inside the “BODY” were remained unchanged.

#### 2.3.2 Image registration and resampling

**FIGURE 3:**
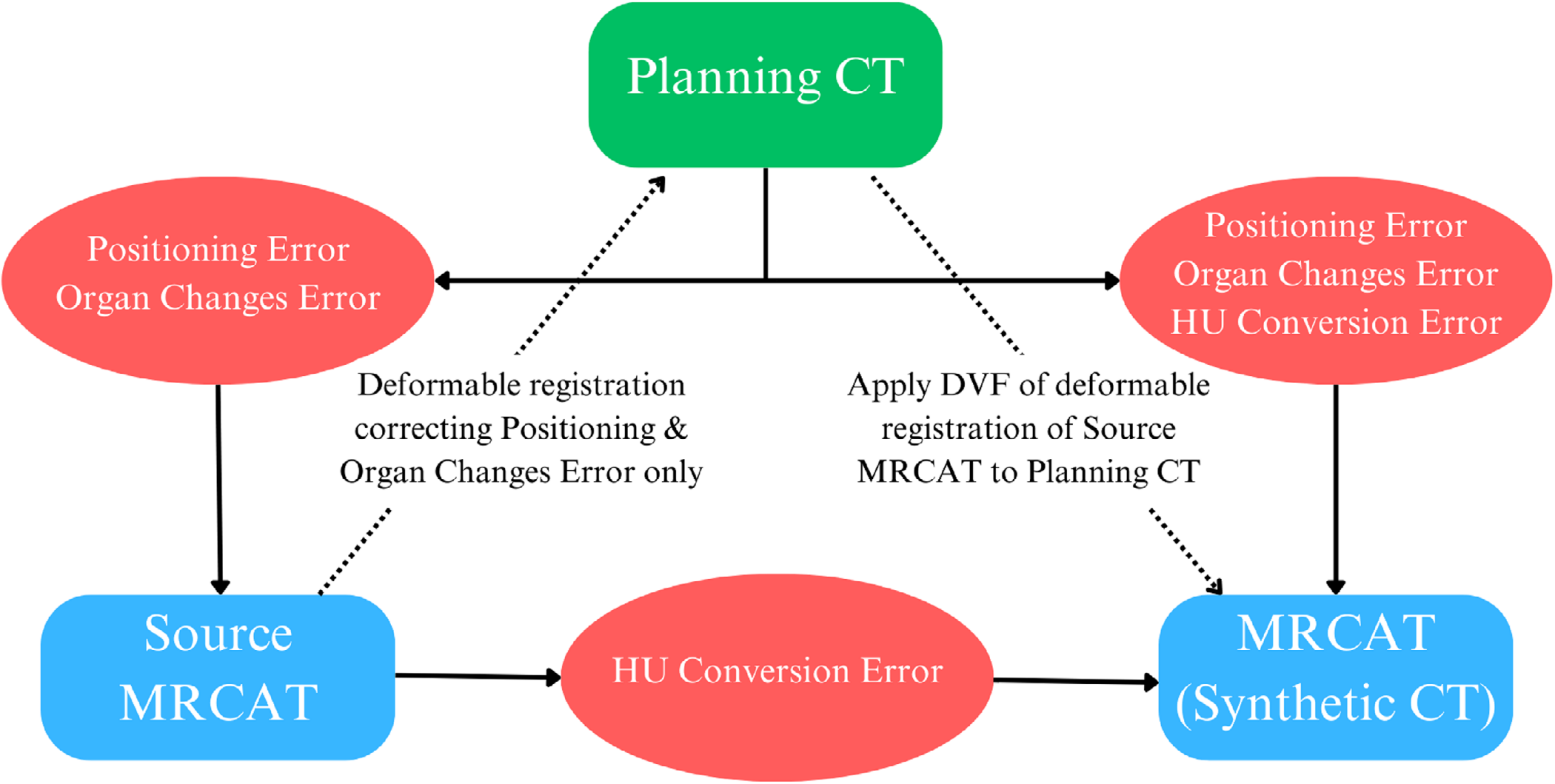
Registration workflow. This chart demonstrated the source of errors (Red boxes and solid arrows) associated between Planning CT (Green box) and images from MR‐simulation (Blue boxes), and the corresponding Pre‐processing procedure performed with rationale explained (Dashed arrows).

The discrepancies in HU between planning CT and synthetic CT were associated with positioning error, organ changes, and accuracy of the MRCAT algorithm. While first two were confounding to be controlled, the last one was the scope of interest for this validation. Instead of direct registration of synthetic CT to planning CT, strategies in Figure 3 have been employed to involve the ground truth image set of source MRCAT (mDIXON) in registration, correcting errors associated with positing and organ motion, while retaining the conversion error of the algorithm to be validated.

For each case, the Source MRCAT (mDIXON), the MRCAT (Synthetic CT), and the planning CT after accessories removal were loaded to the 3D Slicer. Following the deformable registration of the source MRCAT to planning CT using [General Registration (ELASTIX)] module with the [Multimodal Head & Neck] preset, resultant DVF correcting positioning and organ motion errors between two simulation sessions were extracted to files. The MRCAT was then resampled with reference to image origin, voxel dimensions and size of the planning CT, using the corresponding DVF as transform file in [RESAMPLE (BRAIN)] module. The lower range of HU was specified to be −1000 for air to match the planning CT, while the maximum HU was unchanged. Since the DVF for resampling was calculated based on both ground truth images of planning CT and Source MRCAT, resampled MRCAT have corrected errors except error during synthetic CT conversions.

#### 2.3.3 Plan recalculation

The resampled MRCAT, original planning CT, and respective structure sets were imported to the Eclipse (Varian Medical System, California, USA) under the same pseudo‐identity. The registration of planning CT and resampled MRCAT was done to link the geometry and origin between two sets of images in [Registration] module. Since the MRCAT were resampled with reference to the planning CT, zero displacements were made in all dimensions. The structure sets were then copied to the registered resampled MRCAT in the [Contouring] module.

For IMRT/VMAT cases, the resampled MRCAT were assigned as the new structure set for the original treatment plan, and recalculation was then conducted in [External Beam Planning] module using the same calibration curve and the dose calculation model of analytical anisotropic algorithm (AAA). For SRS cases, while original plans were computed with the iPlan (BrainLab, Munich, Germany) using Monte Carlo (MC) algorithms for dose calculation, all original treatment plans were recalculated using the AAA in the Eclipse first, followed by recalculation on respective resampled MRCAT to facilitate comparison with plans of IMRT/VMAT cases.

### 2.4 Data analysis and statistical test

#### 2.4.1 Image quality: Quantitative metrics

Image quality between whole 3D volume of planning CT and synthetic CT were compared using HU‐based metrics, including mean error (ME) that gave direction of deviation, mean absolute error (MAE) that provided attitude of difference between two images, and root mean squared error (RMSE) that enhanced sensitivity to larger error. In addition, indices describing the overall similarity were calculated under normalization within range of [0‐1], including peak signal‐to‐noise ratio (PSNR) that provided authenticity of generated images, and structural similarity index measures (SSIM) that described the geometric distribution of pixels and perceived similarity in terms of luminance, contrast, and structural information. The calculations were performed with in‐house code complied in MATLAB. Metrics for cases of IMRT/VMAT and SRS were tested for normality with Shapiro‐wilk test and compared by independent‐sample *t*‐test with Bonferroni correction was applied based on *p* < 0.05 as statistically significant. Formulae were listed as the following:

(1) $$\mathrm{ME}\;=\frac{1}{n}\;\sum_{i\;=\;1}^{n}\left(\mathrm{sCT}-\mathrm{pCT}\right)\;$$

(2) $$\mathrm{MAE}\;=\;\frac{1}{n}\sum_{i\;=\;1}^{n}\left|\mathrm{sCT}-\mathrm{pCT}\right|$$

(3) $$\mathrm{RMSE}\;=\sqrt{\frac{1}{n}\sum_{i=1}^{n}{\left(\mathrm{sCT}-\mathrm{pCT}\right)}^{2}}\;$$

(4) $$\mathrm{PSNR}\;=\;10\log\left(\frac{sC{\mathrm{Tma}x}^{2}}{\mathrm{MSE}}\right)\;$$

(5) $$\mathrm{SSIM}\;=\frac{\left(2\mu_{\mathrm{sCT}}\mu_{\mathrm{pCT}}+C_{1}\right)\left(2\sigma_{\mathrm{sCT},\mathrm{pCT}}+C_{2}\right)}{\left(\mu_{\mathrm{sCT}}^{2}+\mu_{\mathrm{pCT}}^{2}+C_{1}\right)\left(\sigma_{\mathrm{sCT}}^{2}+\sigma_{\mathrm{pCT}}^{2}+C_{2}\right)}\;$$

For the formulae of calculation, the number of voxels is represented by n in Equations (1) to (3). The HU of voxel in planning CT, synthetic CT, and the maximum HU of synthetic CT are denoted as pCT, sCT, and sCTmax, respectively. For SSIM calculation in Equation (5), the mean HU of synthetic CT and planning CT are expressed as $\mu_{sCT}$ and $\mu_{pCT}$. Standard deviation of HU for synthetic CT, planning CT, and cross‐covariance are $\sigma_{sCT}$, $\sigma_{sCT}$, and $\sigma_{sCT,pCT,}$ respectively. The luminance and contrast comparison constant are represented by $C_{1}$ and $C_{2}$.

#### 2.4.2 Dosimetric parameter: Dose‐volume histogram

The dose distribution for identical plan calculated on planning CT and synthetic CT was extracted and imported into the 3D Slicer. Dose coverage for different structures and OARs were then compared using cumulative DVH parameters that based on dose distributions for each set of CT using the [Dose Volume Histogram] module. Wilcoxon signed rank test was adopted to compare metrics between planning CT and synthetic CT with Bonferroni correction based on *p* < 0.05 as statistically different. The selection of parameters for target volume and OARs have referenced the local dose acceptance specification summarized in Table 2. Despite the difference in acceptance criteria between techniques of IMRT/VMAT and SRS, same set of metrics were adopted to facilitate the comparison.

**TABLE 2:** Brain IMRT/VMAT dose acceptance report criteria for PTV and OARs.

Dose specification for target		Dose specification for OARs	
PTV receiving:	Specification:	OARs	Specification:
100% of prescribed dose	>95% volume	Brainstem	Dmax < 54 Gy
>110% of prescribed dose	<20% volume	Chiasm	Dmax < 54 Gy
<93% of prescribed dose	<1% volume	Bilateral C.N. II	Dmax < 54 Gy
		Bilateral Eyes	Dmax < 54 Gy
		Bilateral Lens	Dmax < 10 Gy
		Bilateral Temporal Lobes	Dmax < 54 Gy < 4cc > 60 Gy
		Bilateral Cochlea & C.N. VIII	Dmean < 50 Gy < 5% > 55 Gy

In PTV coverage, criteria were expressed in the format of percentage volume receiving specific prescribed IL due to discrepancies of dose prescriptions across each patient and diagnosis. Thus, instead of D95% and D99% widely adopted in existing literatures, this study assessed the target coverage in treatment plans calculated on both set of CTs by V93%, V100%, and V110% of prescribed dose.

For OARs constraints, the organ of interest in brain region are brainstem, optic nerve (C.N.II), optic chiasm, eyes, lens, temporal lobes, vestibulocochlear nerve (C.N.VIII), and cochlear, where paired structures with left and right homologues are delineated and regarded as single structure for dose acceptance. Specification in dose report mainly employs the mean dose (D‐mean) or maximum point dose (D‐max) depending on types of organs, and both metrics were recorded for all OARs under the investigation of this study.

#### 2.4.3 Dosimetric agreement: Gamma analysis

Gamma analysis is a quantitative measure tool for verifying the global agreement between two dose distributions and is widely adopted for linear accelerator (LINAC) commissioning and patient‐specific QA in RT technique involves intensity modulations.^23^, ^24^ In practice, dose distribution of treatment plans calculated on TPS would first be superimposed to the dose recorded by phantom after delivering the same plan on LINAC, and sampled with multiple points of measurement. The similarity between the evaluated dose and the reference dose will then be quantified under a set of user‐defined passing criteria, expressed as the distance‐to‐agreement (DTA) and dose difference (DD) at each point.^23^, ^24^ While the DTA specified the physical distance where the evaluated point could find a similar point dose on the reference dose distribution within its ellipsoid, the DD has defined the tolerance of DD between points to be compared on the two doses. Formulae are summarized as follows:

(6) $$\Gamma \;\left(r_{R},r_{E}\right)=\;\sqrt{\frac{\Delta r^{2}\left(r_{R},r_{E}\right)}{\delta r^{2}}+\frac{\Delta D^{2}\left(r_{R},r_{E}\right)}{\delta D^{2}}}$$

(7) $$\gamma \;\left(r_{R}\right)=\;\min\left[\Gamma \left(r_{R},r_{E}\right)\right]\forall \left(r_{E}\right)$$

Equation (6) is defining the pass or fail of the each evaluated point inserted on the superimposed dose distributions, with value ≤ 1 as calculation passes and > 1 as calculation fails. While the actual DD and DTA between two dose distributions are denoted as the $\Delta r(r_{R},r_{E})$ and $\Delta D(r_{R},r_{E})$, the user‐defined passing criteria on DD and DTA are represented by δr and δD. Equation (7) is assessing local passing rates for all evaluated points. Since existence of any evaluated points passing the specified criteria with the reference point in Equation (6) would be regarded as pass and returning the value as < 1, the minimum value would be taken for gamma index in consideration of all evaluated points in Equation (7). Finally, the global passing rate of gamma index for all points of measurement would be assessed, with 95% commonly adopted as clinical acceptance.

The dose distributions calculated on planning CT and synthetic CT were imported to the 3D Slicer and compared using 3D gamma analysis of the [Dose Comparison] module. Dosimetric agreement between each pair of planning CT plan and synthetic CT plan were assessed under the passing criteria of 3% and 3 mm, 2% and 2 mm, and 1% and 1 mm with 10% low‐dose threshold. The gamma passing rate between IMRT/VMAT group and SRS group were also tested for normality with Shapiro‐Wilk test and compared using independent‐sample *t*‐test with Bonferroni correction applied on *p* < 0.05 as statistically significant. Correlation of global passing rate with image quality metrics were tested with Spearman correlation test.

## 3 RESULTS

### 3.1 Quantitative image quality metrics

The ME, MAE, RMSE, PSNR, and SSIM were employed to compare all 33 pairs of planning CT and synthetic CT. Samples of IMRT/VMAT and SRS were calculated separately. The full overlapped range of two CT sets were included as ROI of comparison. All metrics results have followed normal distribution. Generally, deviations between planning CT and synthetic CT for IMRT/VMAT cases were significantly less than that for SRS cases (*p* < 0.001). The IMRT/VMAT and SRS cases were having mean ME of 23.42 ± 1.05 HU with synthetic CT yield higher HU; mean MAE of 38.03 ± 1.42 and 52.36 ± 2.63 HU; mean RMSE of 89.09 ± 6.65 and 108.38 ± 12.23 HU; mean PSNR of 29.11 ± 0.60  and 27.65 ± 0.59 dB; and mean SSIM of 0.88 ± 0.00 and 0.70 ± 0.01, respectively. Results are summarized in Table 3.

**TABLE 3:** Image quality analysis between planning CT and synthetic CT.

	IMRT/VMAT	SRS	*p*
ME (HU)	23.42 ± 1.05	28.39 ± 3.17	<0.001^*^
MAE (HU)	38.03 ± 1.42	52.36 ± 2.63	<0.001^*^
RMSE (HU)	89.09 ± 6.65	108.38 ± 12.23	<0.001^*^
PSNR (dB)	29.11 ± 0.60	27.65 ± 0.59	<0.001^*^
SSIM	0.88 ± 0.00	0.70 ± 0.01	<0.001^*^

Values presented as mean ± standard deviation.

* Statistically significant, independent‐sample *t*‐test.

### 3.2 Dose‐volume histogram parameters

DVH metrics reported were extracted after recalculation in Eclipse. For target volume coverage in 18 IMRT/VMAT cases, volume‐based metrics of V_93%IL_, V_100%IL_, and V_110%IL_ are included in Table 4. In comparing V_93%IL_ and V_100%IL_, no significant differences were identified between plans calculated on planning CT and synthetic CT. For V_110%IL_, 32 cases were having 0% volume coverage for both plans with only one case having 0.08% deviation, and thus, statistical test was not applicable. V_prescribed IL_ was evaluated for 15 SRS cases with different prescribed IL, and no significant difference in target coverage was identified after recalculation.

**TABLE 4:** Dosimetric comparison of PTV coverage between planning CT and synthetic CT.

Target volume	Metrics	Planning CT (%)	Synthetic CT (%)	Difference (%)	Normalized difference (%)	*p*
IMRT /VMAT	V_93%IL_	99.65 ± 0.54	99.60 ± 0.54	−0.05 ± 0.11	−0.05 ± 0.11	0.25
	V_100%IL_	97.13 ± 1.29	96.80 ± 1.34	−0.32 ± 0.46	−0.34 ± 0.47	0.02
	V_110%IL_	0.01 ± 0.02	0.001 ± 0.01	−0.005 ± 0.02	−4.40 ± 18.12	NA
SRS	V_prescribed IL_	96.58 ± 2.04	96.48 ± 2.16	−0.10 ± 0.62	−0.11 ± 0.64	0.93

Values presented as mean ± standard deviation.

*Statistically significant, Wilcoxon Signed Rank Test.

In review of OAR constraints, certain contours of selected cases were unavailable, including locally advanced tumor with margin undifferentiable from normal structures, postoperative cases with the eyeball excised, or with cases with treatment target located far from the selected structures. Thus, number of samples varied with the selected OARs, and statistical test for temporal lobes was inapplicable due to small number of samples. The D‐mean and D‐max between plans calculated on planning CT and synthetic CT were extracted and compared in Table 5. All OARs for SRS were tested with no significant differences in doses across CT sets. While similar results of no significant deviations were obtained between plans of IMRT/VMAT, D‐mean of the left lens was 0.70% higher (*p* < 0.001) after recalculation on synthetic CT.

**TABLE 5:** Dosimetric comparison of OAR dose between planning CT and synthetic CT.

		IMRT/VMAT						SRS					
OAR	Metric	n	Planning CT (Gy)	Synthetic CT (Gy)	Difference (Gy)	Normalize difference (%)	*p*	n	Planning CT (Gy)	Synthetic CT (Gy)	Difference (Gy)	Normalize difference (%)	*p*
Brain‐stem	D‐mean	18	22.12 ± 13.46	22.13 ± 13.47	0.01 ± 0.06	0.24 ± 0.63	0.53	14	0.33 ± 0.47	0.33 ± 0.46	0.00 ± 0.00	0.04 ± 0.66	0.11
	D‐max		40.93 ± 17.25	40.94 ± 17.25	0.02 ± 0.12	0.19 ± 0.49	0.88		1.70 ± 2.60	1.70 ± 2.59	−0.00 ± 0.01	0.04 ± 0.66	0.83
Chiasm	D‐mean	17	29.33 ± 15.05	29.33 ± 15.03	0.01 ± 0.09	0.25 ± 0.58	0.59	11	0.28 ± 0.62	0.28 ± 0.62	0.00 ± 0.00	−0.01 ± 0.67	0.42
	D‐max		38.00 ± 18.64	38.03 ± 18.61	0.04 ± 0.14	0.52 ± 1.70	0.59		0.47 ± 1.13	0.47 ± 1.13	0.00 ± 0.00	−0.16 ± 0.63	0.60
Left C.N. II	D‐mean	18	18.10 ± 12.97	18.12 ± 12.99	0.02 ± 0.08	0.37 ± 0.93	0.35	13	0.32 ± 0.69	0.32 ± 0.69	−0.00 ± 0.00	−0.66 ± 3.02	0.70
	D‐max		26.92 ± 15.58	26.94 ± 15.60	0.02 ± 0.11	0.25 ± 0.62	0.31		0.55 ± 1.36	0.54 ± 1.37	−0.01 ± 0.04	−3.72 ± 13.70	0.47
Right C.N. II	D‐mean	18	20.20 ± 15.57	20.22 ± 15.59	0.02 ± 0.07	0.38 ± 0.91	0.10	13	0.32 ± 0.88	0.32 ± 0.88	−0.00 ± 0.01	−0.69 ± 2.06	0.81
	D‐max		28.84 ± 17.66	28.87 ± 17.66	0.03 ± 0.11	0.28 ± 0.61	0.20		0.56 ± 1.52	0.56 ± 1.52	−0.01 ± 0.03	−0.92 ± 3.28	0.56
Left Eye	D‐mean	18	9.08 ± 5.37	9.10 ± 5.37	0.02 ± 0.03	0.53 ± 1.36	0.01	15	0.13 ± 0.20	0.13 ± 0.20	0.00 ± 0.00	0.47 ± 1.50	0.06
	D‐max		20.77 ± 14.68	20.76 ± 14.67	−0.01 ± 0.05	0.26 ± 1.18	0.98		0.46 ± 0.94	0.46 ± 0.93	0.00 ± 0.01	0.68 ± 1.74	0.54
Right Eye	D‐mean	18	10.02 ± 6.31	10.02 ± 6.30	0.00 ± 0.03	0.43 ± 1.39	0.40	15	0.16 ± 0.40	0.16 ± 0.40	0.00 ± 0.00	0.49 ± 1.72	0.28
	D‐max		23.27 ± 16.16	23.26 ± 16.14	−0.01 ± 0.05	0.24 ± 1.18	0.35		0.61 ± 1.77	0.61 ± 1.77	0.00 ± 0.00	0.440 ± 1.40	0.21
Left Lens	D‐mean	18	4.68 ± 1.93	4.70 ± 1.94	0.02 ± 0.03	0.70 ± 1.31	<0.001^*^	15	0.10 ± 0.16	0.10 ± 0.16	−0.00 ± 0.00	0.03 ± 2.60	0.28
	D‐max		6.05 ± 2.37	6.07 ± 2.37	0.02 ± 0.03	0.62 ± 1.33	0.03		0.16 ± 0.26	0.16 ± 0.26	−0.00 ± 0.01	−0.04 ± 2.90	0.36
Right Lens	D‐mean	18	4.78 ± 2.04	4.80 ± 2.04	0.01 ± 0.03	0.59 ± 1.39	0.13	15	0.09 ± 0.17	0.09 ± 0.16	−0.00 ± 0.01	0.40 ± 4.59	0.36
	D‐max		6.21 ± 2.46	6.22 ± 2.46	0.01 ± 0.04	0.49 ± 1.42	0.23		0.15 ± 0.29	0.142 ± 0.29	−0.00 ± 0.01	−0.21 ± 3.24	0.69
Left Temp‐oral	D‐mean	8	27.73 ± 21.87	27.67 ± 21.79	−0.06 ± 0.09	0.01 ± 0.49	NA						
	D‐max		45.91 ± 18.39	45.86 ± 18.32	−0.05 ± 0.16	−0.03 ± 0.36	NA						
Right Temp‐oral	D‐mean	6	13.67 ± 8.84	13.65 ± 8.77	−0.02 ± 0.05	−0.03 ± 0.47	NA						
	D‐max		39.91 ± 9.02	39.82 ± 9.00	−0.10 ± 0.06	−0.25 ± 0.15	NA						
Left Coch‐lea	D‐mean	16	15.06 ± 12.53	15.05 ± 12.51	−0.02 ± 0.06	0.09 ± 0.74	0.38						
	D‐max		19.20 ± 17.42	19.23 ± 17.46	0.02 ± 0.13	0.22 ± 0.81	0.57						
Right Coch‐lea	D‐mean	15	18.65 ± 16.17	18.62 ± 16.18	−0.03 ± 0.09	−0.20 ± 0.41	0.21						
	D‐max		22.17 ± 18.60	22.17 ± 18.65	−0.00 ± 0.10	−0.16 ± 0.34	0.28						
Left C.N. VIII	D‐mean	15	16.28 ± 14.56	16.28 ± 14.55	−0.01 ± 0.06	0.09 ± 0.49	0.8						
	D‐max		21.95 ± 19.52	21.98 ± 19.56	0.03 ± 0.09	0.17 ± 0.58	0.46						
Right C.N. VIII	D‐mean	14	18.95 ± 15.29	18.94 ± 15.29	−0.01 ± 0.07	−0.04 ± 0.28	0.30						
	D‐max		24.32 ± 19.27	24.35 ± 19.31	0.03 ± 0.12	0.09 ± 0.36	0.44						

Values presented as mean ± standard deviation.

* Statistically significant, Wilcoxon Signed Rank Test.

### 3.3 Gamma analysis

Calculation of gamma indices for 33 cases was performed as shown in Table 6. Global passing rates for IMRT/VMAT and SRS were 99.92% and 99.86% in 3%/3 mm; 99.42% and 99.52% in 2%/2 mm; and 96.47% and 97.57% in 1%/1 mm. While the passing rate has been decreasing from 3%/3 mm to 1%/1 mm due to the stringent criteria and lower tolerance in difference of both DD and DTA, passing rates are higher than the clinical acceptance of 95% in 3%/3 mm even when assessing with criteria of 1%/1 mm. In addition, no statistical differences were identified between passing rate of cases between IMRT/VMAT and SRS for all three criteria.

**TABLE 6:** Gamma analysis between planning CT and synthetic CT for IMRT/VMAT and SRS cases.

Criteria	IMRT/VMAT	SRS	*p*
3%/3 mm	99.92 ± 0.12	99.86 ± 0.27	0.53
2%/2 mm	99.42 ± 0.55	99.52 ± 0.55	0.46
1%/1 mm	96.47 ± 2.34	97.57 ± 1.64	0.07

Values presented as mean ± standard deviation.

^*^Statistically significant, independent‐sample *t*‐test.

### 3.4 Correlation test between image quality and gamma passing rate

Spearman's rank correlation coefficients were calculated between metrics of ME, MAE, RMSE, PSNR, and SSIM; and the gamma passing rate under criteria of 3%/3 mm, 2%/2 mm, and 1%/1 mm in Table 7. The MAE and RMSE, as negatively oriented index, have been significantly correlated with IMRT/VMAT cases under all gamma passing criteria with −0.50 and −0.55 on 3%/3 mm (*p* = 0.03; *p* = 0.02), −0.71 and −0.74 on 2%/2 mm (*p* < 0.01; *p* < 0.01), and −0.63 and −0.66 on 1%/1 mm (*p* = 0.01; *p* < 0.01) respectively. The PSNR, as a positively oriented index, was significantly correlated with IMRT/VMAT cases under all gamma passing criteria with 0.49 on 3%/3 mm (*p* = 0.04), 0.72 on 2%/2 mm (*p* < 0.01), and 0.57 on 1%/1 mm (*p* = 0.01). The ME and SSIM were not significantly correlated to all samples under any gamma passing criteria. Meanwhile, SRS cases were not significantly correlated with any given image quality metrics.

**TABLE 7:** Correlation test between image quality metrics and global passing rate of gamma analysis.

	IMRT/VMAT					SRS				
Gamma passing criteria	ME	MAE	RMSE	PSNR	SSIM	ME	MAE	RMSE	PSNR	SSIM
3%/3 mm	0.02 [0.94]	−0.50^*^ [0.03]	−0.55^*^ [0.02]	+0.49^*^ [0.04]	+0.41 [0.10]	−0.17 [0.54]	−0.06 [0.84]	0.10 [0.73]	−0.19 [0.49]	−0.02 [0.93]
2%/2 mm	0.24 [0.34]	−0.71^*^ [< 0.01]	−0.74^*^ [< 0.01]	+0.72^*^ [< 0.01]	+0.41 [0.09]	−0.03 [0.90]	−0.23 [0.42]	−0.02 [0.95]	−0.02 [0.95]	0.05 [0.85]
1%/1 mm	0.12 [0.76]	−0.63^*^ [0.01]	−0.66^*^ [< 0.01]	+0.57^*^ [0.01]	+0.40 [0.10]	0.11 [0.69]	−0.16 [0.58]	−0.06 [0.84]	0.04 [0.89]	−0.14 [0.62]

Value represented are the Spearman's Rank Correlation Coefficient (rS).

+ve, Positive correlation; ‐ve, Negative correlation.

* *p* < 0.05, Spearman correlation test.

## 4 DISCUSSION

MR‐simulation has been prompting interest from the community due to its variability in soft tissue contrast and capability of functional imaging that facilitate target delineation and OARs contouring for enhancing the treatment outcome. Meanwhile, high‐quality synthetic CT is an essential component for dose calculation following individualized treatment planning. The results of this study have successfully validated the clinical use of the MRCAT synthetic CT algorithm in RT planning for brain tumors with reconcilable results in terms of image quality and dosimetric agreement to the current standard of planning CT and previous validations.^7^, ^20^

**FIGURE 4:**
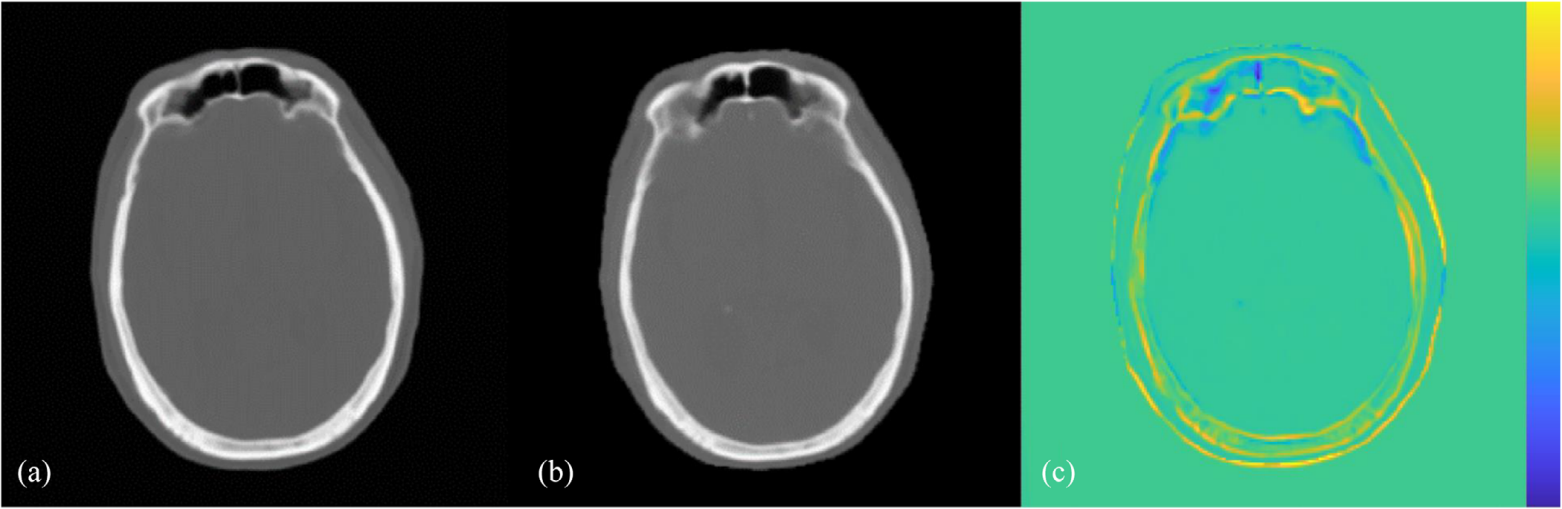
Axial image for subject with highest MAE among IMRT/VMAT samples. This demonstrated that area with high HU deviation were locating as bony interfaces surrounding with tissues and air. The Resampled MRCAT (a) was subtracted with planning CT (b) to obtain subtraction map (c) with yellow and blue colour contributed from resampled MRCAT and planning CT, respectively.

Concerning image quality analysis, ME and MAE are the widely adopted metrics to evaluate the absolute HU difference between planning CT and synthetic CT. ME from exiting studies concerning head and neck region synthetic CT ranged from 6.7 to 37.5 HU,^25^, ^26^ agreed with the 23.42 ± 1.05 and 28.39 ± 3.17 HU for IMRT/VMAT and SRS cases. Meanwhile, the typical range of MAE documented for brain region deep learning algorithms ranged from 44 to 100 HU,^27^, ^28^, ^29^, ^30^, ^31^, ^32^, ^33^, ^34^, ^35^, ^36^ slightly higher than the mean MAE of 38.03 ± 1.42 HU for IMRT/VMAT cases, and reconcilable to that of 52.36 ± 2.63 HU for SRS cases obtained from this validation. While the results implied a superior performance of MRCAT brain, the outlying MAE should be interpreted with cautions. As the error‐based metrics including MAE have averaged the difference of all pixels for calculation, the larger ROI selection tends to yield a better result due to increased proportion of surrounding air with less HU difference included. In addition, regions with highest MAE were focused on bony interface with soft tissues and intra‐cranial cavities as demonstrated in Figure 4. Echoed similar studies of synthetic CT,^7^, ^17^ ^,^ ^19^a common challenge for synthetic CT algorithms is the short T2 relaxation time of cortical bone, resulting in ambiguous interface with adjacent compartments.

**FIGURE 5:**
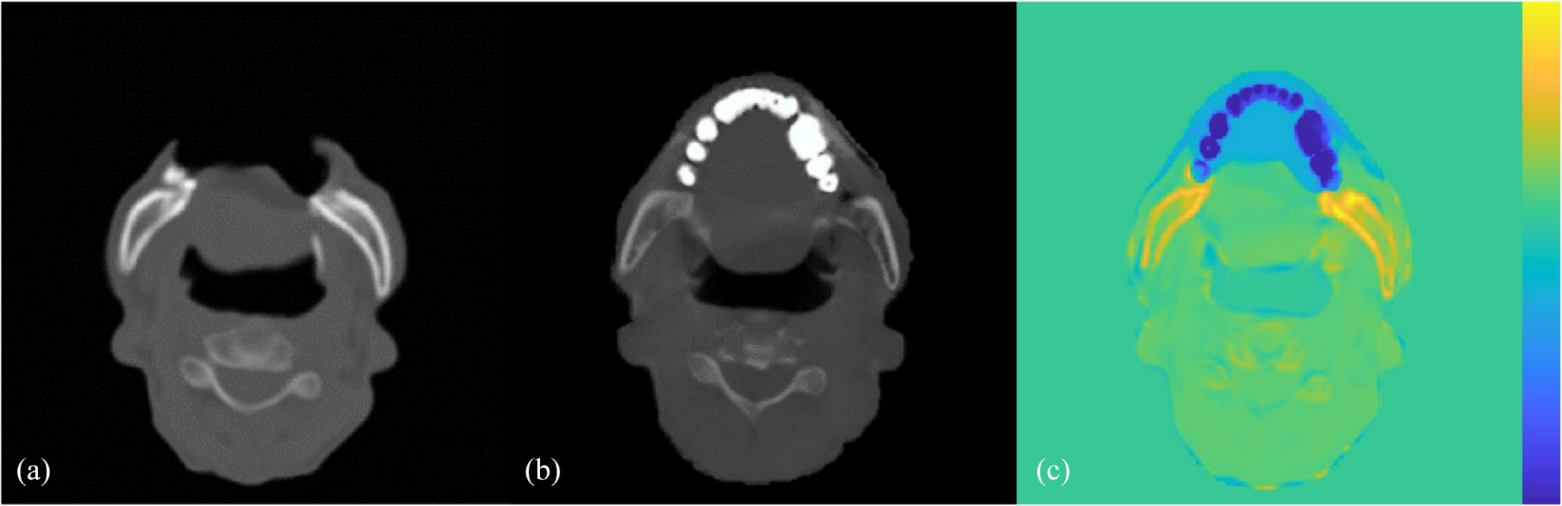
Axial image for subject with fixed denture. This showed the effect of susceptibility artifact to synthetic CT generation. The resampled MRCAT (a) was subtracted with planning CT (b) to obtain subtraction map (c) with yellow and blue colour contributed from resampled MRCAT and planning CT respectively.

The RMSE and PSNR have both penalized larger deviations from reference images of synthetic CT. The values reported in existing literatures were largely varied across the site of interest and network architectures adopted for training, in which the algorithms dedicated for brain regions were ranged from 24.2 to 32.4 dB,^28^, ^34^, ^35^, ^36^, ^37^ and also encompassed the present results 29.11 ± 0.60  and 27.65 ± 0.59 dB for IMRT/VMAT and SRS cases, respectively. Aside from the precision of algorithm, disparities across studies might be attributed to artifacts of dentures or surgical implants. Since PSNR lack spatial account of pixel intensities distribution and largely dependent on maximum HU across all pixels in each slice, it might not capture localized or spatially varying artifacts within the images. As shown in Figure 5, dentures or metallic implants with ferromagnetic nature could induce local field inhomogeneity, resulting in susceptibility artifact with signal void attributed to accelerated T2* dephasing. This might be the reason for Yuan et al.^38^ obtained outlying results from similar studies by the low RMSE of 10−45 HU and PSNR up to 49−51 dB, as all samples with metallic artifacts were excluded.

**FIGURE 6:**
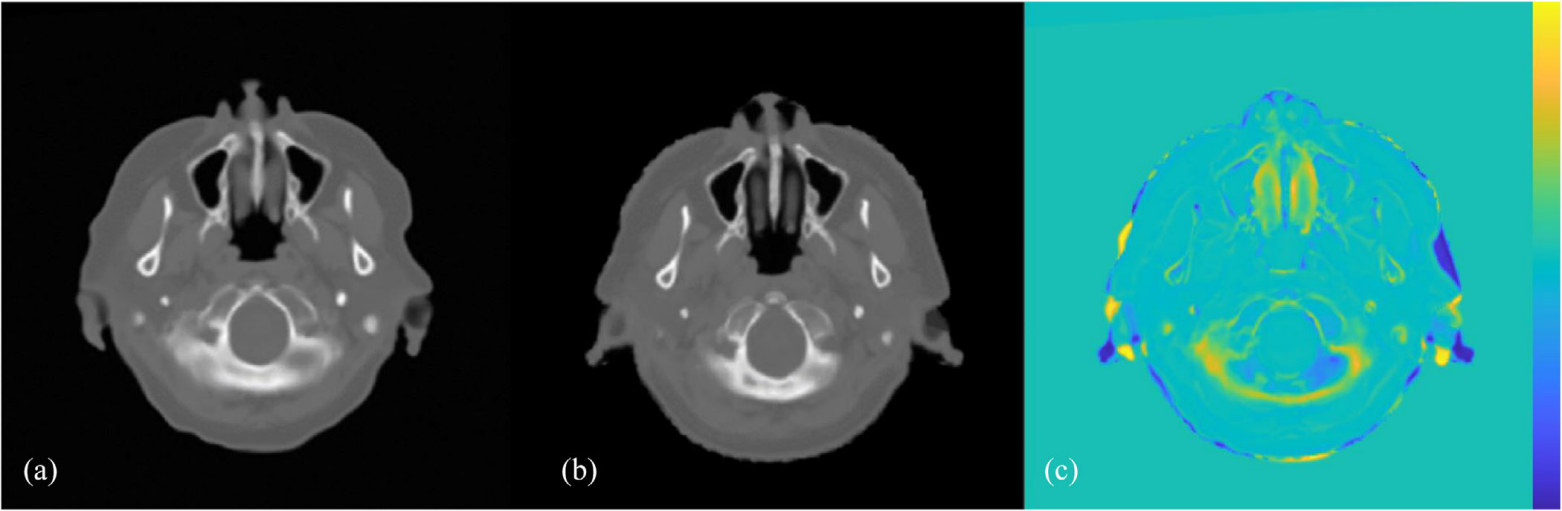
Axial image for sample with highest MAE and RMSE. This showed the effect of facial contour distortion by immobilization device. The resampled MRCAT (a) was subtracted with planning CT (b) to obtain subtraction map (c) with yellow and blue colour contributed from resampled MRCAT and planning CT, respectively.

Unlike quantitative error‐based metrics, SSIM measured geometric distribution and perceptual similarity between images accounting luminance, contrast, and structural information. For the studies with algorithm trained for brain region, Li et al.^28^ have recorded SSIM of 0.91–0.94 with multiple deep‐learning architectures, while Massa et al.^31^ have presented results ranged from 0.61 to 0.64 with U‐Net based on conventional MRI sequences and with fat saturation techniques. Although the presented results have been encompassed by existing literatures, SRS cases with mean SSIM of 0.70 were having significantly lower similarity than IMRT/VMAT cases with mean SSIM of 0.88. The sample with highest deviation was presented in Figure 6, showing the facial contour distortion by the stereotactic mask in Planning CT, which absent in MR‐simulation, resulting in uncorrectable error even with deformable registration. However, correlation test has not established correlation between SSIM and dosimetric accuracy, as dose was calculated based on attenuation estimation by HU rather than structural information.

Aside from SSIM, higher similarity of synthetic CT to planning CT in IMRT/VMAT than SRS cases were also recorded in other image quality metrics, which might potentially be attributed to treatment setup reproducibility. For IMRT/VMAT cases, identical immobilization of Type‐S system was adopted in CT‐ and MR‐simulation. In contrast, the MR‐compatible version of stereotactic mask used for SRS cases was unavailable, compromising the position accuracy of synthetic CT. Despite the clinical indication of synthetic CT is for dose calculation only, the geometric error inherited from the unreproduced treatment position might also affect the image of MR‐simulation, which are indicated for target and OAR localization. Thus, ensuring the availability of MR‐compatible setup device is suggested prior to clinical implementation.

**FIGURE 7:**
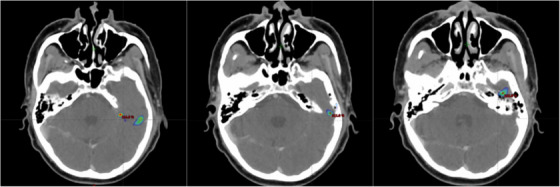
Isodose color wash for subject IM011 with 110%IL lower threshold. These slices of color wash demonstrated the discrepancies of V110% between plans after recalculation were contributed by the area located near bony structures.

Dosimetrically, volume‐based DVH metrics of V93%, V100%, and V110% for IMRT/VMAT; and Vprecsribed IL for SRS cases were adopted to normalize differences in prescribed dose and IL between cases and techniques. Existing studies mostly adopted dose‐based metrics of D95% and D99% for measuring difference after recalculation on synthetic CT. Algorithms for head and neck were reported to have 0.7–1.6% PTV dose deviation,^25^, ^26^ whereas varied results have been obtained in brain region studies. While Gupta et al.^27^ recorded 2.3% differences for D‐mean with U‐net trained algorithm, Alvarez Andres et al.^30^ and Tang et al.^39^ have reported deviation down to 0.3% and 0.13%, respectively. The MRCAT Brain have also obtained FDA clearance with less than 1% doses deviation for PTV coverage as compared to planning CT.^40^ Although direct comparison is hindered, reconcilable results with 0.01–1.04% differences in the target volume irradiation were measured in this study. For V93%IL and 100% IL, no significant difference was identified with average volume difference of 0.3%, reflected a promising agreement of lower constraints and radical dose coverage. For V110%IL, most cases have recorded 0% for both plans, indicated a promising agreement in high‐dose regions. Isodose color wash in Figure 7 has presented an outlier with 0.08% absolute difference in 110%IL coverage, where deviation was located superior to the left petrous ridge, echoed the limitation due to short T2 of bone as mentioned. The 0.11% volume difference in prescribed dose of SRS cases has further validated a favurable agreement even for small field dosimetry.

**FIGURE 8:**
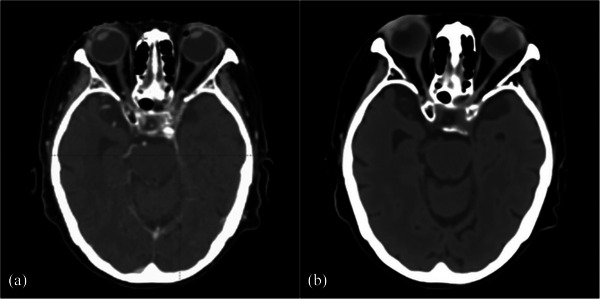
Axial CT slices for subject IM013. This comparison showed that both eyes of the patient panned toward the right side during CT‐simulation (a) and MR‐simulation (b) in different extent.

For OAR constraints, unlike pelvis synthetic CT studies, D‐max supplemented with D‐mean was adopted for serial OARs in brain region. Among synthetic CT for head and neck region, Dinkla et al.^41^ and Qi et al.^42^ have both reported less than 1% differences with algorithms of U‐Net and cGAN, while Klages et al.^25^ have recorded OAR dose deviation up to 1.7–2.0%. Reduced difference has been recorded in brain region, where Liu et al.^29^ and Tang et al.^39^ have reported less than 1.5% and 0.77% respectively. The results for MRCAT brain in this study were generally consistent with previously documented in literatures, having 0.01–0.69% percentage difference among all samples, without statistically significant difference identified after recalculation on for most OARs, except for the D‐mean of left lens. With the increased acquisition time, unintended motions of eyeball are more likely to happen in MR‐simulation, and consequently changed the lens position compared to CT‐simulation as the outlier shown in Figure 8. Since the smaller size of lens has only captured in few slices with original dose is down to few Gy, slight deviation could result in prominent percentage difference after recalculation as recorded to be 0.7% for presented samples, which is 0.02 Gy in actual and may not results in presentable difference of clinical outcome.

**FIGURE 9:**
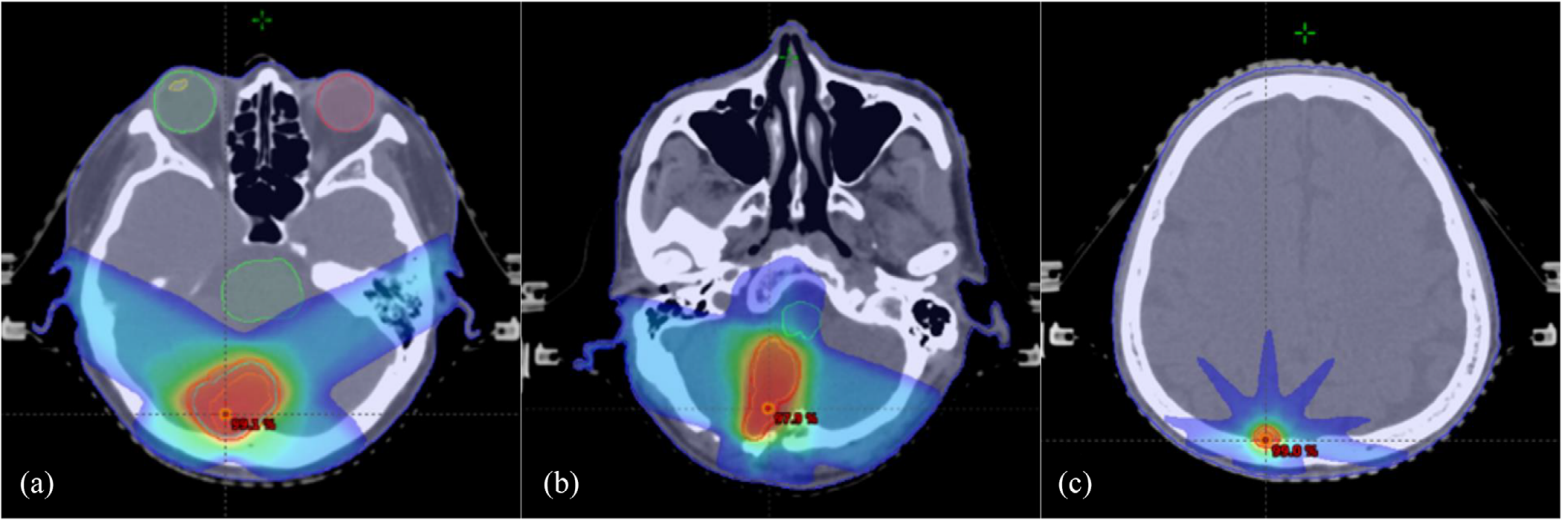
Isodose color wash showing locations of Isocenter for three outliers in the SRS. Target located near occipital bone (a), near cerebellum (b), and near posterior boundary of skull (c).

Gamma analysis provided a global assessment of dosimetric agreement after plan recalculation. In studies employing cGAN for training brain region algorithms, Koike et al.^43^ recorded passing rates of 95.3%, 99.2%, and 99.8% for 1%/1 mm, 2%/2 mm, and 3%/3 mm, while Kazemifar et al.^32^ also reached passing rate of 94.6% and 99.2% for 1%/1 mm and 2%/2 mm, respectively. Comparable results were also reported from Alvarez Andres et al.^30^ using High ResNet to achieve 97.9%, 99.6%, and 99.8% for 1%/1 mm, 2%/2 mm, and 3%/3 mm, respectively. Comparing to our results, all cases have reached the clinical acceptance of 95% passing rate under criteria of 3%/3 mm, and comparable to most deep‐learning algorithms. In criteria of 1%/1 mm, 2%/2 mm, and 3%/3 mm, IMRT/VMAT cases were recorded 96.5% ± 2.3%, 99.4% ± 0.6%, and 99.9% ± 0.1%, while SRS cases also reached 97.5% ± 1.6%, 99.5 ± 0.5%, and 99.8% ± 0.3% accordingly. For SRS, most cases have reached 100% passing rate in 3%/3 mm with three exceptions of 99.23%, 99.29%, and 99.73% as outlined in Figure 9. Targets for these cases were located at cerebellar region or occipital lobe, where target were closed to or surrounded by bone‐soft tissue interface and expected with compromised synthetic CT accuracy. Since such phenomenon was not observed in IMRT/VMAT cases, the effects were likely to be the combined effect of small target volume for SRS cases, and the small field dosimetry uncertainties for AAA. In addition, all gamma passing criteria in SRS cases were not correlated to any metrics. Since the main contributor of compromised image similarity in SRS is the immobilization as discussed, results have demonstrated the image quality may not even be an effective indicator of dosimetric accuracy when treatment immobilization and setup were not reproduced.

Despite the comparable results obtained, several practical considerations should be given before its clinical application. First, availability of MR‐compatible immobilization should be checked. As image quality analysis and correlation test have established the importance of setup reproducibility, ensuring identical simulation device including the MR‐compatible version of instruments is essential to reduce positioning error for accurate dose calculation. Second, treatment technique adopted for the case should be considered. SRS cases with lower error tolerance due to highly conformed dose and tight treatment margin might not be suitable candidate for MR‐only planning. This yielded further study performed with preproduced setup and on MC dose calculation algorithm. Third, the target located or extended near boundaries between bone and soft tissues should be excluded. The misclassification of compartment and HU assignation located at bony interface have contributed to most outliers in our data analysis, which might result in inaccurate dose assumptions during planning, and consequently causing under‐ or overdose to target or adjacent OARs. Investigation of UTE sequences or other correction algorithms might be considered to improve the account of bone with short T2 relaxation in the synthetic CT.

## 5 CONCLUSION

The present study has validated the clinical implementation of synthetic CT for dose calculation and possibility of MR‐only planning workflow by using MRCAT brain for brain tumor RT. Resampled synthetic CT has demonstrated comparable similarity in terms of image quality with previous deep learning algorithms, provided that identical treatment setup was reproduced. Recalculation of treatment plans on synthetic CT is also observed with consistent dosimetric accuracy and agreement with other validation studies based on selected DVH metrics for target and OARs, and gamma analysis with criteria of 3%/3 mm, 2%2 mm, and 1%/1 mm. Given the significant correlation of image quality and dosimetric accuracy, the MR‐simulation with the MRCAT brain algorithm should be considered as the standalone simulation modality for RT planning under the premises of reproduced immobilization. Systematic case selections and address of practical considerations are essential before actual clinical implementation.

## AUTHOR CONTRIBUTIONS

**Tyrone Tsz Yeung Yip**: Conceptualization; methodology; data collection; data analysis; and writing. **Zhichun Li**: Data pre‐processing. **Tian Li**: Professional and academic supervision. All authors have read and agreed to the published version of the manuscript.

## CONFLICT OF INTEREST STATEMENT

The authors declare no conflicts of interest.
